# MFHAS1 Is Associated with Sepsis and Stimulates TLR2/NF-κB Signaling Pathway Following Negative Regulation

**DOI:** 10.1371/journal.pone.0143662

**Published:** 2015-11-24

**Authors:** Jing Zhong, Qi-Qing Shi, Min-Min Zhu, Jian Shen, Hui-Hui Wang, Duan Ma, Chang-Hong Miao

**Affiliations:** 1 Department of Anesthesiology, Fudan University Shanghai Cancer Center; Department of Oncology, Shanghai Medical College, Fudan University, Shanghai, China; 2 Children’s Hospital of Fudan University, Shanghai, China; 3 Key Laboratory of Molecular Medicine, Ministry of Education, Department of Biochemistry and Molecular Biology, Institutes of Biomedical Sciences, School of Basic Medical Sciences, Fudan University, Shanghai, China; University of Torino, ITALY

## Abstract

Malignant fibrous histiocytoma amplified sequence 1 (MFHAS1) has a potential immunoregulatory role dependent on Toll-like receptors (TLRs). TLR2, associated with deleterious systemic inflammation, cardiac dysfunction, and acute kidney injury, acts synergistically in sepsis. The role of MFHAS1 in targeting TLR2 involved in sepsis has not been examined thus far. This study aimed to examine the relationship of MFHAS1 and sepsis, and the effect of MFHAS1 on the TLR2 signaling pathway. Blood samples were collected from eight sepsis patients after surgery and eight patients undergoing selective surgery to determine blood MFHAS1 levels. HEK 293 cells, RAW 264.7 macrophages and THP-1 monocytes were used to confirm the effect of MFHAS1 on TLR2 signaling pathway. Our study showed that blood MFHAS1 was significantly elevated in septic patients, and MFHAS1 was more increased in mononuclear cells from septic patients. Pam3CSK4 (TLR2 ligand) was found to induce MFHAS1 production in RAW 264.7 murine macrophages and THP-1 human monocytes in a time-dependent manner. MFHAS1 has dual effects on TLR2 signaling pathway and inflammation, i.e., inhibitory effect at 6 hours, and then stimulatory effect after 24 hours through the activation of TLR2/NF-κB signaling pathway, and MFHAS1 induced the phosphorylation of JNK and p38 after TLR2 stimulation.

## Introduction

MFHAS1 (malignant fibrous histiocytoma amplified sequence 1) is a member of the ROCO protein family, which consists of multi-domain proteins sharing a conserved ROC-COR supra-domain [1, 2]. MFHAS1 is a predicted oncoprotein in MFHs (malignant fibrous histiocytomas) and gastrointestinal tumors [1, 3, 4]. It can also regulate erythropoiesis through the Raf/MEK/ERK pathway [5]. Interestingly, Ng et al. found that siRNA knockdown of *Mfhas1* in RAW 264.7 macrophages strongly enhances IL-6 production following LPS and polyI:C stimulation, suggesting a potential immunomodulatory role for MFHAS1 [6]. MFHAS1 was found to regulate the transmembrane Toll-like receptor (TLR)–dependent signaling [6].

Modulation of TLR signaling as a therapeutic strategy is an area of research that has prompted much excitement and debate in the management of sepsis. Sepsis is an important cause of mortality [7–9], and is the most common cause of mortality in the intensive care unit (ICU) [10]. Death from sepsis is understood to be a complex process, driven by a lack of normal immune homeostatic functions, and an excessive production of proinflammatory cytokines, which leads to multi-organ failure. To improve clinical management and outcomes in critically ill patients, the Surviving Sepsis Campaign guidelines were published ten years ago, and were last revised in 2012 [11]. However, to date, there have been no commercially available successful strategies for targeting the molecular pathways involved in sepsis.

The vast majority of sepsis cases involve infection with either gram-positive or gram-negative bacteria [12, 13]. The innate immune system forms the first line of defense against microbial infection, and is activated by the engagement of innate immune receptors, also known as pattern recognition receptors (PRRs), in response to invading pathogenic microbes [14–16]. TLRs, with an extracellular domain involved in bacterial ligand recognition, are the most widely described PRRs [17–20]. Eleven TLRs have been discovered in humans [19]. TLR2 and TLR4, which are expressed on the cell surface, are the only TLRs known to be responsive to microbial ligands [21]. TLR4 and TLR2 signaling is the key pathway in sepsis pathophysiology [22].

Our previous studies (unpublished) have shown, by real-time fluorescence quantitative PCR, that the relative mRNA levels of MFHAS1 in *TLR4* knockout mice are lower than that in wild type mice, and that MFHAS1 may play a negative regulatory role in TLR4 signaling pathway. Ng et al. studied TLR4 and TLR3 [6]. Unlike TLR4and TLR3, TLR2 is associated with deleterious systemic inflammation, cardiac dysfunction, acute kidney injury (a common entity in critically ill patients) and is mostly triggered by severe sepsis [23–25]. TLR2 is a functional receptor for components of gram-positive bacteria including LTA, peptidoglycan (PGN), and bacterial lipopeptides, thus being responsible for the detection of gram-positive bacteria [26, 27]. TLR2 also plays a critical role in mediating mitochondrial dysfunction in peritoneal leukocytes, during polymicrobial sepsis [28]. In humans, signaling by TLR2 activates the MyD88 pathway, which results in the activation of transcription factors, including nuclear factor-κB (NF-κB), members of the interferon (IFN)-regulatory factor (IRF) family, and mitogen-activated protein kinase (MAPK) signaling pathways, which ultimately leads to the production of inflammatory cytokines, C reactive protein (CRP), Pentraxin 3,etc. MAPKs include p38, c-Jun NH2-terminal kinase (JNK), and extracellular signal-related kinase 1/2 (ERK1/2) [29]. IL-6, and CRP, etc. serve as biomarkers, which may aid clinical decision-making and predicting sepsis-related outcomes [30–32].

Molecules targeting TLR signaling pathways in sepsis exist, but thus far none have proven efficacy in clinical trials [20]. Clinical trials with biomodulators aimed at inhibiting inflammatory signaling pathways have generally failed to improve outcomes in patients with sepsis [33–35]. MFHAS1 is expected to act as a new biomodulator, affecting TLRs signaling. Until now, little was known about the relationship of MFHAS1 and sepsis, and the mechanisms underlying the effect of MFHAS1 on the TLR2 signaling pathway and inflammation. The aim of this present study is to investigate the relationship of MFHAS1 and sepsis, and the effects of MFHAS1 on the TLR2 signaling pathway and inflammation, and highlight this protein as an optional target in the treatment of inflammation in sepsis.

## Methods

### Patients and blood samples

Patients who were admitted to the ICU for sepsis after surgery at Fudan University Shanghai Cancer Center between January 2014 and September 2014 were enrolled in our study. The 2012 Surviving Sepsis Campaign guidelines were used to diagnose sepsis [36]. Patients who were admitted to the ICU after surgery not for sepsis at Fudan University Shanghai Cancer Center between January 2014 and September 2014 were enrolled in the control group. All procedures were performed under a protocol approved by the Ethics Committee at Fudan University Shanghai Cancer Center, and written informed consent was obtained from all patients. The methods were carried out in accordance with the approved guidelines. A total of sixteen patients (eight patients with sepsis and eight patients without sepsis) were included. We collected data regarding age, gender, types of disease, and types of infection. Blood samples were collected from patients after including in the study. Each blood sample was transferred immediately to a heparinized tube and centrifuged at -80°C. Blood samples were used to measure the concentrations of IL-6 and MFHAS1 through ELISA.

### Isolation of peripheral blood mononuclear cells (PBMC)

PBMC were isolated from human collected blood samples by density gradient centrifugation over Lympholyte Mammal (TBDscience, China) according to the instructions of the manufacturer. Briefly, blood was diluted 1:1 with RPMI-1640 medium (*v/v*), precisely applied on the surface of the gradient (density: 1.086 ± 0.001 g/cm^3^) and centrifuged at 400 ×* g* for 30 min at 15°C. Then, the PBMC layer at the interface was carefully collected and transferred into a 15 ml Falcon tube, washed with RPMI-1640 medium and centrifuged again for 10 min at 800 ×* g*. The obtained cell pellet was re-suspended in RPMI-1640 medium for western blotting.

### Reagents and chemicals

Pam3CSK4 (TLR2 ligand) and Lipofectamine 2000 were purchased from InvivoGen® (San Diego, CA, USA), anti–GAPDH and anti-HIS mAbs were purchased from Abcam® (Cambridge, UK), anti-pJNK (phosphorylated JNK), anti–JNK, anti-pERK (phosphorylated ERK), anti-ERK, anti-pp38 (phosphorylated p38), anti-p38, anti-p-p65 (phosphorylated p65) mAbs were purchased from Cell Signalling Technology® (Danvers, MA, USA), anti-MFHAS1 (K-19) polyclonal antibody was purchased from Santa Cruz Biotechnology, Inc.® (Dallas, TX, USA).

### Cells

The HEK-293 cell line with stable *MFHAS1* expression (referred to as “293-MFHAS1” in this article), human acute monocytic leukemia cell line (THP-1), RAW 264.7 macrophage were all provided by Dr. Miao (Fudan University Shanghai Cancer Center, China) as a gift. Cells were maintained in high-glucose DMEM (HyClone, Thermo, USA) supplemented with penicillin, streptomycin, and 10% FBS (HyClone).

### Plasmids

pGL4.32[*luc2P*/NF-κB–RE/Hygro] and renilla vector reporter plasmid were purchased from Promega® (Madison, USA), REPO^TM^AP-1 vector reporter plasmid and IRF7-Gal4 fusion vectors were purchased from GenomeDitech® (Shanghai, China), expression vectors for human TLR2 and monocyte differentiation antigen CD14 (CD14) were purchased from Genechem® (Shanghai, China). Plasmid pCDH-shMFHAS1 was gifted by Dr. Miao.

### Luciferase assay

Transfections for luciferase assays were carried out using the HEK 293 cell line. Subconfluent HEK 293 cells were transfected with 10 ng renilla (internal transfection control), 100 ng pGL4.32[*luc2P*/NF-κB–RE/Hygro] vector reporter plasmid (firefly luciferase), or 20 ngREPO^TM^AP-1 vector reporter plasmid (firefly luciferase), 100ng pCDH-TLR2 with or without 50 ng CD14 expression plasmids, and empty vector, for an equal amount of DNA in each well. For the IRF7 assays, 1–3 ng IRF7-Gal4 fusion vectors were used in combination with 60 ng pFR luciferase reporter. All the transfections were completed using Lipofectamine 2000. At 24 h post-transfection, cells were treated with Pam3CSK4 (100 ng/mL or 10μg/mL). Dual-Luciferase Reporter Assay System (Promega, Madison) was used to test luciferase activity. Firefly luciferase activity was normalized to renilla luciferase activity.

### RNA isolation and quantitative RT-PCR

The cells and their growth media were collected. The cells were collected in Trizol® (Invitrogen, Carlsbad, CA) to isolate total RNA, according to the manufacturer’s instructions. RNA 200 ng was used as a template in reverse transcription with iScript™ cDNA Synthesis Kit (Bio-Rad, Hercules, CA). cDNA was amplified using primers specific for *IL6* and *Mfhas1*. Real-time PCR was performed with the iTaq™ SYBR Green Supermix (Bio-Rad, Hercules, CA) in the StepOnePlus™ Real-Time PCR System (Life Technologies, USA). GAPDH and β-actin was used to normalize the amount of RNA in samples. Primers used in this study can be found in S1 Table.

### ELISA

Cytokine levels were measured in cell supernatant (without FBS) by ELISA, performed according to the manufacturer’s instructions. We used the following ELISA kits: mouse IL-6 (R&D Systems, Inc. Minneapolis, USA), mouse TNF-α (R&D Systems, Inc. Minneapolis, USA), and human IL-6 (Proteintech, Chicago, USA). Human blood MFHAS1 ELISA kit was purchased from CUSABIO® (Wuhan, China).

### Western blotting

Samples containing equal amounts of protein were fractionated on a 10% SDS-PAGE gel and transferred onto a Hybond TM-P membrane (GE Healthcare, Little Chalfont, UK) by using Trans-Blot cell (Bio-Rad Laboratories, Hercules, CA, USA). The membrane was then blocked with blocking solution (8% skim milk in TBS-T, according to vendor’s suggestion) at room temperature for 1 h, followed by incubation with titrated primary-antibody-containing blocking solution (5% BSA in TBS-T) at 4°C overnight. On the second day, the blot was washed three times with 10 ml TBS-T for 15 min, and then incubated with titrated HRP conjugated secondary antibody in blocking solution (5% BSA in TBS-T) for 1 h. After TBS-T washes (5 min, three times), the target protein signals on the membrane were visualized by chemiluminescence reagent (Merck Millipore, Billerica, MA, USA) exposed on X-ray films (MidSci, St. Louis, MO, USA). For image signal quantification, the scanned gel TIFF files were further analyzed by using Image J software.

During the assessment of pJNK, pp38, pERK, phosphatase inhibitor and protease inhibitor were used in cell lysis. pp38 was first tested by western blotting, and then p38, pERK, ERK, pJNK, JNK were successively tested after stripping for 20 min using the Rapid Stripping Buffer (Promoton, Shanghai, China).

### Statistical analysis

GraphPad Prism Version 5 (GraphPad Software, San Diego, CA) was used to compare two or more means by *t*-test, one-way or two-way ANOVA, depending on the experimental conditions. When an overall statistically significant difference was measured (*p* < 0.05), a Tukey post-test was performed to adjust the *P* value for multiple comparisons. The *P* values, and the respective comparison for which they were calculated, are indicated in the figure legends. Data are expressed as means ± SD. *P* values below 0.05 were considered significant. All experiments were performed in triplicate, and equivalent results were obtained in each experiment.

## Results

### MFHAS1 is elevated in septic patients and in macrophages/monocytes following TLR2 stimulation

We recruited eight septic patients (all patients were gram-positive infection), post-surgery, from the ICU. All patients were diagnosed as having sepsis, based on the Surviving Sepsis Campaign guidelines 2012. Eight patients in the control group were admitted to the ICU after surgery not for sepsis. The gender, ages, types of disease, and types of infection did not differ significantly between sepsis and control groups (*p* > 0.05). Blood IL-6 and MFHAS1 concentrations were found to be significantly higher in the sepsis group than in the control group (*p* < 0.05) (Table 1).

**Table 1:** Gender, age, blood IL-6 and MFHAS1 concentrations in septic patients and controls.

Group	Gender	Age	IL-6 (pg/mL)	MFHAS1(pmol/L)
Sepsis (*n* = 8)	M(80%)	63.67±5.28	35.06±8.09	819.00±135.09
Control (*n* = 8)	M(60%)	63.17±7.17	4.63±1.32	531.17±129.16
*P*	0.490	0.894	<0.0001	0.004

**Fig 1:**
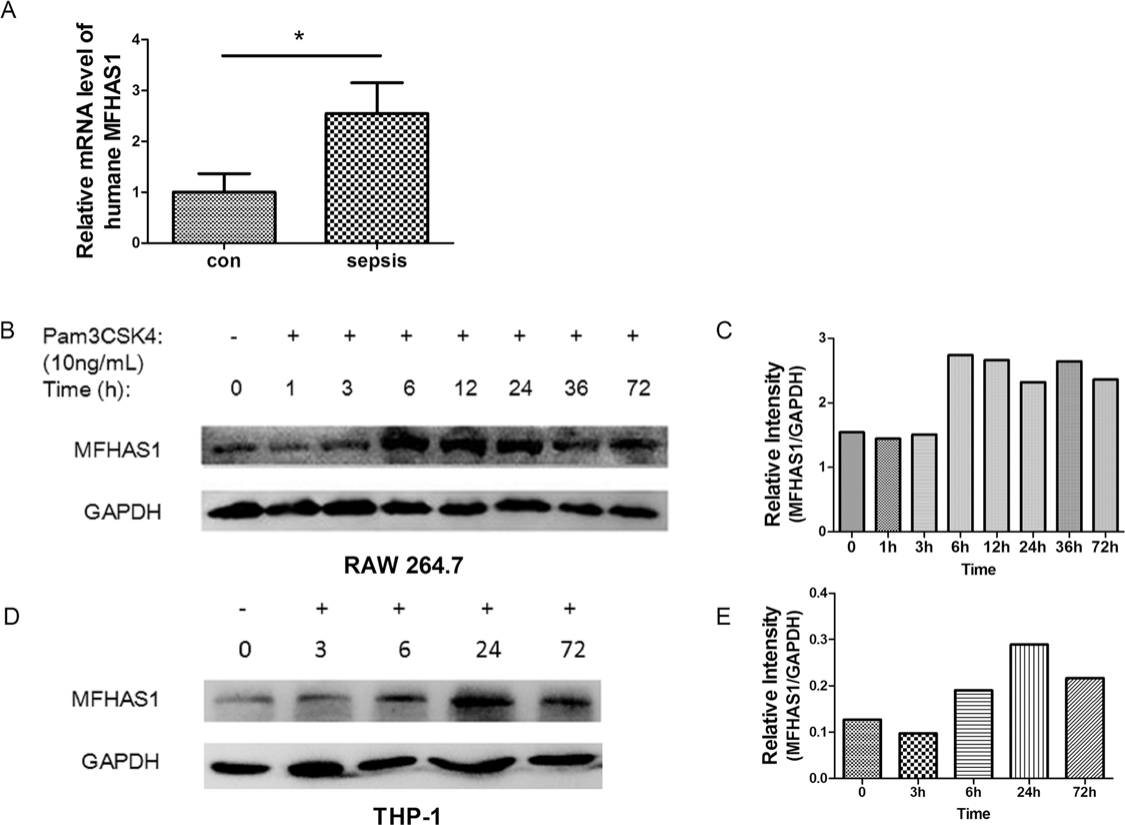
The expression of *MFHAS1* in PBMC and macrophages/monocytes after TLR2 stimulation. (A) PBMC was isolated from the peripheral blood of humane in the control group and the septic patients. The gene expression of *MFHAS1* in the PMBC was analyzed by qPCR. n = 8/group. (B) After stimulation with 10 ng/mL Pam3CSK4 for designated time, RAW 264.7 macrophages indicated a time lag in *Mfhas1* expression. The expressions of *MFHAS1* and *GAPDH* were detected by western blotting. (C) The corresponding optical density of MFHAS1 bands normalized with GAPDH. (D) After stimulation with 10ng/mL Pam3CSK4 for designated time, THP-1 indicated a time lag in *MFHAS1* expression. The expressions of *MFHAS1* and *GAPDH* were detected by western blotting. (E) The corresponding optical density of MFHAS1 bands normalized with GAPDH. Data are presented as mean ± SD in each group. Image J was used in optical density measurement otherwise as indicated. *p < 0.05, compared with control.

*MFHAS1* mRNA was found to be more increased in PBMC from septic patients than control ones (p = 0.032) (Fig 1A). TLR2 ligand, Pam3CSK4, induces MFHAS1 production in RAW 264.7 and THP-1 cells, in a time-dependent manner, until 72 h. The expression of *Mfhas1* increased significantly at 6 h stimulation, and continues to remain high after 24 h, however, it does not show further elevation after this time point (Fig 1B, 1C, 1D and 1E).

### MFHAS1 has an inhibitory effect on TLR2 and inflammation after 6 hours stimulation

**Fig 2:**
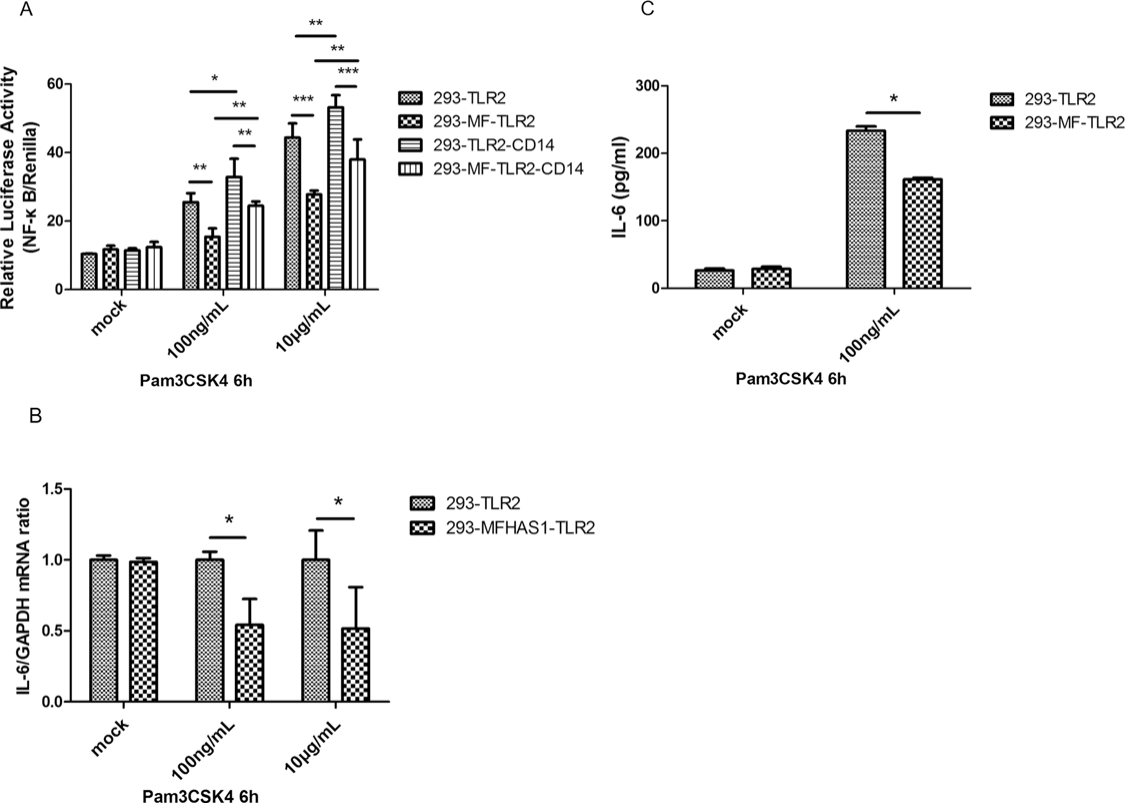
MFHAS1 inhibits the transcriptional activity of NF-κB and IL-6 production 6 h after stimulation with Pam3CSK4 through TLR2. (A) HEK 293 cells and 293-MFHAS1 cells were transiently transfected with 100 ng TLR2 or 100 ng TLR2/50 ng CD14 expression plasmids, and 100 ng NF-κB-dependent luciferase reporter plasmid as well as 10 ng renilla plasmid. Post transfection for 24 h, these transfected cells were exposed to mock treatment, Pam3CSK4 100 ng/mL or 10 μg/mL for 6 h, and fold increase in luciferase activity was measured for NF-κB activation using dual luciferase kits. The relative luciferase activity was calculated from the ratio of NF-κB-Luc (firefly) activity to renilla activity. (B) HEK 293 cells and 293-MFHAS1 cells were transiently transfected with 100 ng TLR2, and 24 h post-transfected cells were untreated or exposed to Pam3CSK4 100 ng/mL or 10μg/mL. After treatment for 6 h, IL-6 expression was assayed by quantitative RT-PCR and normalized to GAPDH. (C) HEK 293 cells and 293-MFHAS1 cells were transiently transfected with 100 ng TLR2, and 24 h post-transfected cells were untreated or exposed to Pam3CSK4 100 ng/mL. The cell supernatant was collected and the amounts of IL-6 were determined by ELISA at 6 h post-treatment. Values are the means ± SD from at least three independent experiments. *p < 0.05, **p < 0.01, or ***p < 0.001.

We used Pam3CSK4 to stimulate TLR2 for 6 h, and found that the relative luciferase activity of NF-κB versus renilla was significantly lower in 293-MFHAS1 cells transiently transfected with TLR2 or TLR2/CD14 than in the control cells (HEK 293 cells transiently transfected with TLR2 or TLR2/CD14) (*p* < 0.01) (Fig 2A), and induction of IL-6 expression was also significantly lower in 293-MFHAS1 cells than in the control cells, as measured by RT-PCR and ELISA (*p* < 0.05) (Fig 2B and 2C). These results demonstrate that MFHAS1 inhibits TLR2 signaling pathway and inflammation at the first 6 hours. CD14 and the elevation of Pam3CSK4 concentration can assist TLR2 to further enhance the activation of NF-κB (*p* < 0.05) (Fig 2A). The efficiency of transfection of TLR2 plasmids does not differ significantly between groups, as measured by western blotting (S1 Fig).

### MFHAS1 has a stimulatory effect on the TLR2 and inflammation, after 24 hours stimulation

**Fig 3:**
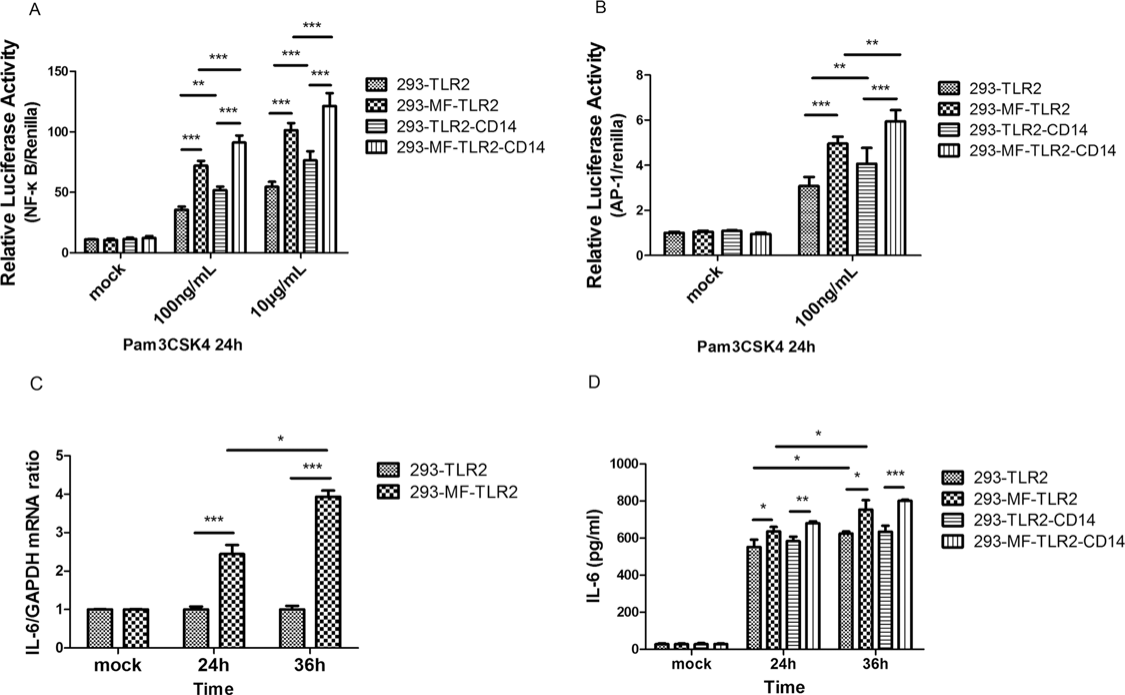
MFHAS1 activates NF-κB, AP-1, and IL-6 expression 24 h after stimulation with Pam3CSK4 through TLR2. (A, B) HEK 293 cells and 293-MFHAS1 cells were transiently transfected with 100 ng TLR2 or 100 ng TLR2/50 ng CD14 expression plasmids, an100 ng NF-κB luciferase reporter plasmid (A) or 20 ng AP-1 luciferase reporter plasmid (B) and 10 ng renilla plasmid. 24 h post-transfected cells were exposed to mock treatment, Pam3CSK4 100 ng/mL or 10μg/mL. At 24 h posttreatment, fold increase in luciferase activity was measured for NF-κB or AP-1 activation using dual luciferase kits. The relative luciferase activity was calculated from the ratio of NF-κB/AP-1 (firefly) activity to renilla activity. (C, D) HEK 293 cells and 293-MFHAS1 cells were transiently transfected with 100 ng TLR2 or 100 ng TLR2/50 ng CD14 expression plasmids, and 24 h post-transfected cells were untreated or exposed to Pam3CSK4 100 ng/mL. After 24 h and 36 h posttreatment, induction of IL-6 expression was assayed by quantitative RT-PCR and normalized to β-actin (C). Cell supernatant was collected and the amounts of IL-6 were determined by ELISA (D). Values are the means ± SD from at least three independent experiments. *p < 0.05, **p < 0.01, or ***p < 0.001.

The relative luciferase activities of NF-κB versus renilla, and AP-1 versus renilla, were both found to be significantly higher in 293-MFHAS1 cells than in the control cells after 24 h stimulation with Pam3CSK4, through TLR2 (*p* < 0.001). CD14 and the elevation of Pam3CSK4 concentration can assist TLR2 to further enhance the activation of NF-κB and AP-1 (*p* < 0.01) (Fig 3A and 3B). Induction of IL-6 expression was also significantly higher in 293-MFHAS1 cells than in control cells, as measured by RT-PCR and ELISA after 24 h stimulation with Pam3CSK4, through TLR2 (*p* < 0.05). Induction of IL-6 expression measured by RT-PCR and ELISA at 36 h was significantly higher than that at 24 h (*p* < 0.05) (Fig 3C and 3D).

**Fig 4:**
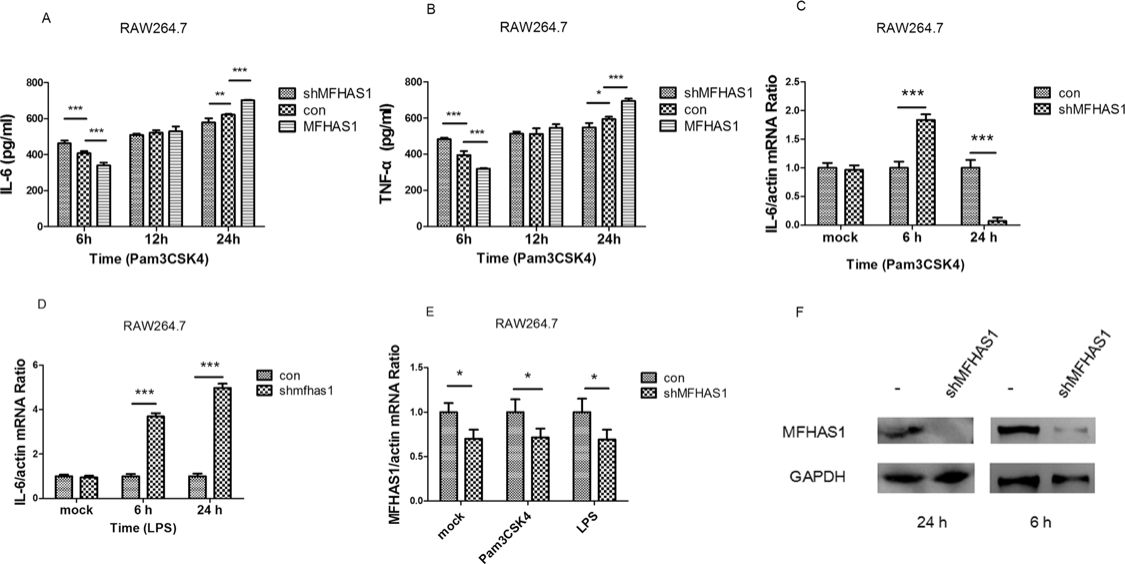
IL-6 and TNF-α production after stimulation with Pam3CSK4 by knockdown of *Mfhas1* using shMFHAS1. (A, B) RAW 264.7 macrophages were transfected with shMFHAS1 or pCDH empty vector or HIS-MFHAS1 plasmid, and 24 h post-transfected cells were treated with 10 ng/mL Pam3CSK4. Cell supernatant was collected after 6, 12, and 24 h posttreatment, and IL-6 (A) and TNF-α (B) production were measured by ELISA. (C, D) RAW 264.7 macrophages were transfected with shMFHAS1 or pCDH empty vector, and 24 h post-transfected cells were untreated or treated with 10 ng/mL Pam3CSK4 (C), or 50 ng/mL LPS (D). At 6 h and 24 h posttreatment, IL-6 expression was measured by RT-PCR and normalized to β-actin. (E, F) The knockdown efficiency of shMFHAS1 at 6 h and 24 h was assessed by RT-PCR (E) and western blotting (F). Values are the means ± SD from at least three independent experiments. *P < 0.05.

Knockdown using shMFHAS1 directed against *Mfhas1* in RAW 264.7 macrophages significantly activated IL-6 and TNF-α production, following 6 h stimulation with Pam3CSK4, through TLR2, as measured by ELISA (*p* < 0.001). However, when RAW 264.7 macrophages in which knockdown had been performed using shMFHAS1 were stimulated with Pam3CSK4 for 24 h, through TLR2, IL-6 and TNF-α production both significantly declined (*p* < 0.05) (Fig 4A and 4B). The RT-PCR results of IL-6 were the same as those with ELISA (Fig 4C). However, MFHAS1 has different effect on TLR4. Following 6 and 24 h stimulation with LPS, through TLR4, knockdown using shMFHAS1 directed against *Mfhas1* in RAW 264.7 macrophages significantly activated IL-6 production (*p* < 0.001) (Fig 4D). The levels of secreted IL-6 and TNF-α production following 6 h Pam3CSK4 stimulation, as measured by ELISA, were significantly lower in RAW 264.7 macrophages transfected with HIS-MFHAS1 (264.7-MFHAS1) than in control cells (*p* < 0.001). However, when cells were stimulated for 24 h, the levels of secreted IL-6 and TNF-α production were both significantly higher in 264.7-MFHAS1 than in control cells (*p* < 0.001). Moreover, IL-6 and TNF-α production rose in a time-dependent manner (*p* < 0.05) (Fig 4A and 4B). The efficiency of shMFHAS1 knockdown was confirmed by both RT-PCR and western blotting (Fig 4E and 4F).

### MFHAS1 does not affect IRF-7 and IFN-β production

**Fig 5:**
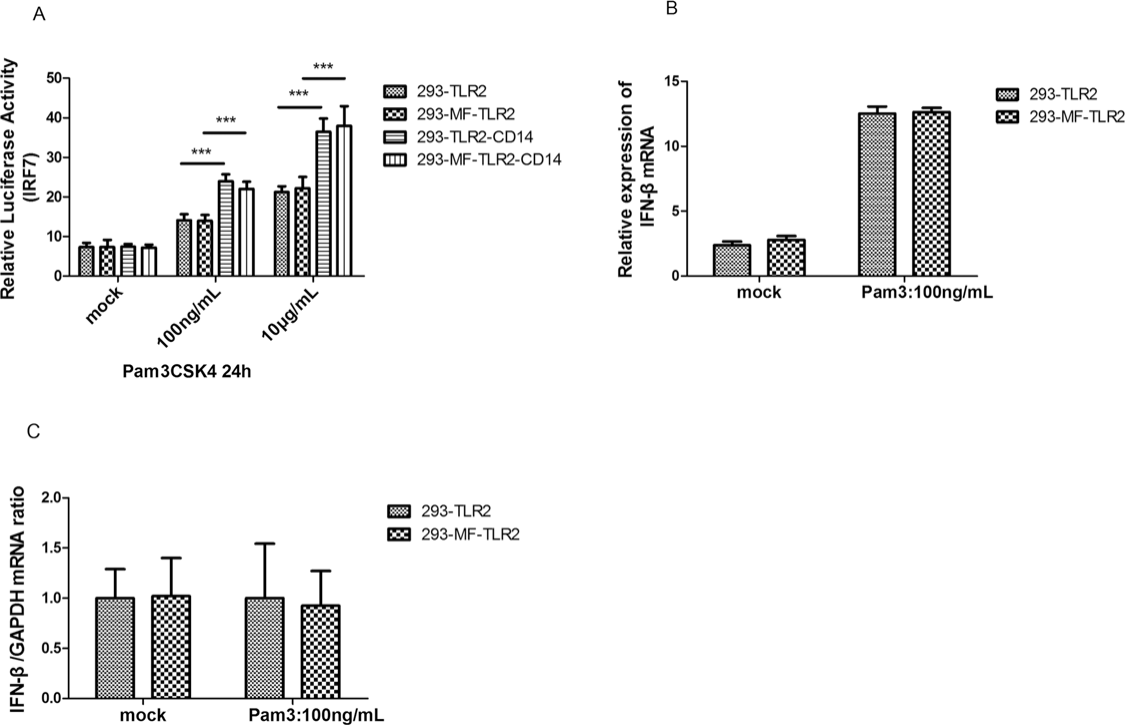
MFHAS1 does not affect transcription factor IRF-7 and IFN-β expression. (A) HEK293 and 293-MFHAS1 cells were transiently transfected with 100 ng TLR2 or 100 ng TLR2/50 ng CD14 expression plasmids, the pFR luciferase reporter gene along with plasmid expressing IRF7-Gal4 3 ng. Then these 24 h post-transfected cells were treated with mock, 100 ng/mL or 10μg/mL Pam3CSK4 for 24 h, and luciferase reporter gene activity was measured. (B) HEK293 and 293-MFHAS1 cells were transiently transfected with 100 ng TLR2 expression plasmids, and 24 h post-transfected cells were untreated or treated with 100 ng/mL Pam3CSK4 for 24 h. IFN-β mRNA expression was assayed by quantitative RT-PCR. (C) The relative IFN-β mRNA level was normalized to GAPDH. Values are the means ± SD from at least three independent experiments. *p < 0.05, **p < 0.01, or ***p < 0.001.

Signaling by TLR2 in humans involves adaptor proteins, which interact with downstream protein kinases that ultimately lead to the activation of transcription factors, including members of the IRF family apart from NF-κB. No significant differences were observed in luciferase activity in transcription factor IRF-7 (Fig 5A) and inflammatory factor IFN-β production (Fig 5B and 5C) between 293-MFHAS1 transiently transfected with TLR2 or TLR2/CD14 and HEK 293 cells transiently transfected with TLR2 or TLR2/CD14 (control) after 24 h stimulation with Pam3CSK4 through TLR2 (*p* > 0.05). However, CD14 and an increase in Pam3CSK4 concentration can assist TLR2 to further enhance the activation of IRF-7 (*p* < 0.05) (Fig 5A).

### MFHAS1 induces the phosphorylation of JNK and p38 in a time-dependent manner

**Fig 6:**
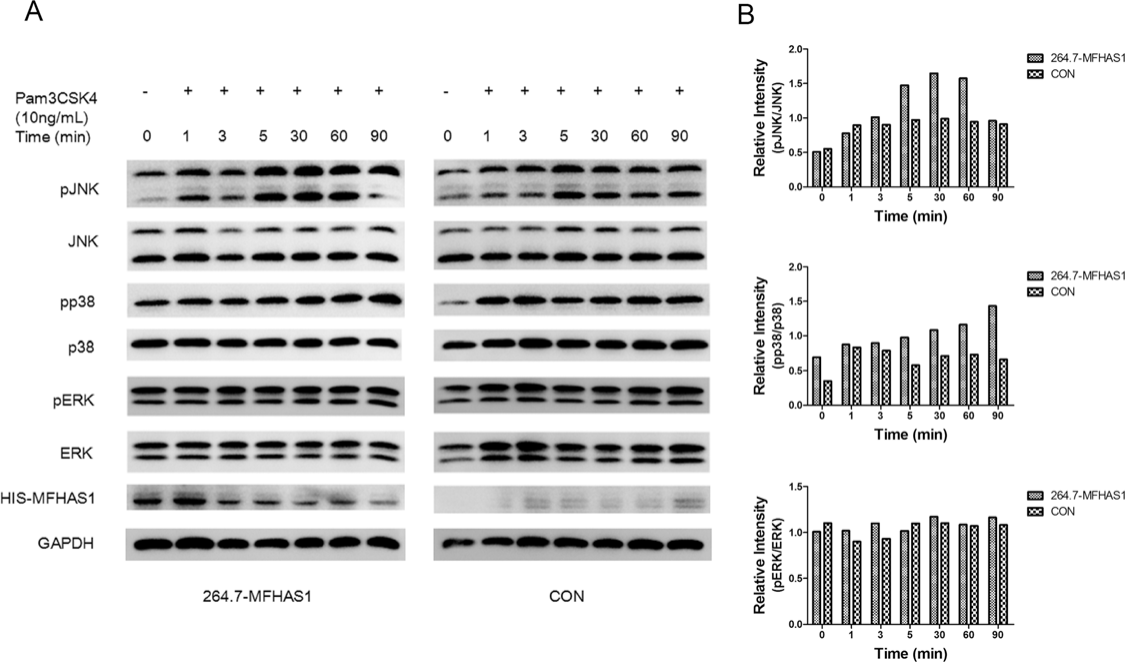
MFHAS1 induces phosphorylation of JNK and p38. (A) RAW 264.7 macrophages were transfected with HIS-MFHAS1 or pCDH empty vector plasmid, and 24 h post-transfected cells were treated with 10 ng/mL Pam3CSK4 for 0, 1, 3, 5, 30, 60, 90 min, respectively. Cells were collected and the protein levels of pJNK, JNK, pp38, p38, pERK, ERK, HIS-MFHAS1 and GAPDH were examined by western blotting. (B) Quantified data of the pJNK, pp38, pERK levels. Levels of pJNK, pp38, pERK respectively normalized to JNK, p38, ERK levels were shown.

The expression of pJNK was higher in 264.7-MFHAS1 than in control cells, immediately (5 min) following stimulation with 10 ng/mL Pam3CSK4 until 1 h. The expression of pp38 was also higher in 264.7-MFHAS1 than in the control cells after 5 min stimulation with 10 ng/mL Pam3CSK4. However, pERK levels did not increase earlier in 264.7-MFHAS1 than in control cells. These results demonstrate that MFHAS1 induces the phosphorylation of JNK and p38, but not ERK, in a time-dependent manner (Fig 6).

**Fig 7:**
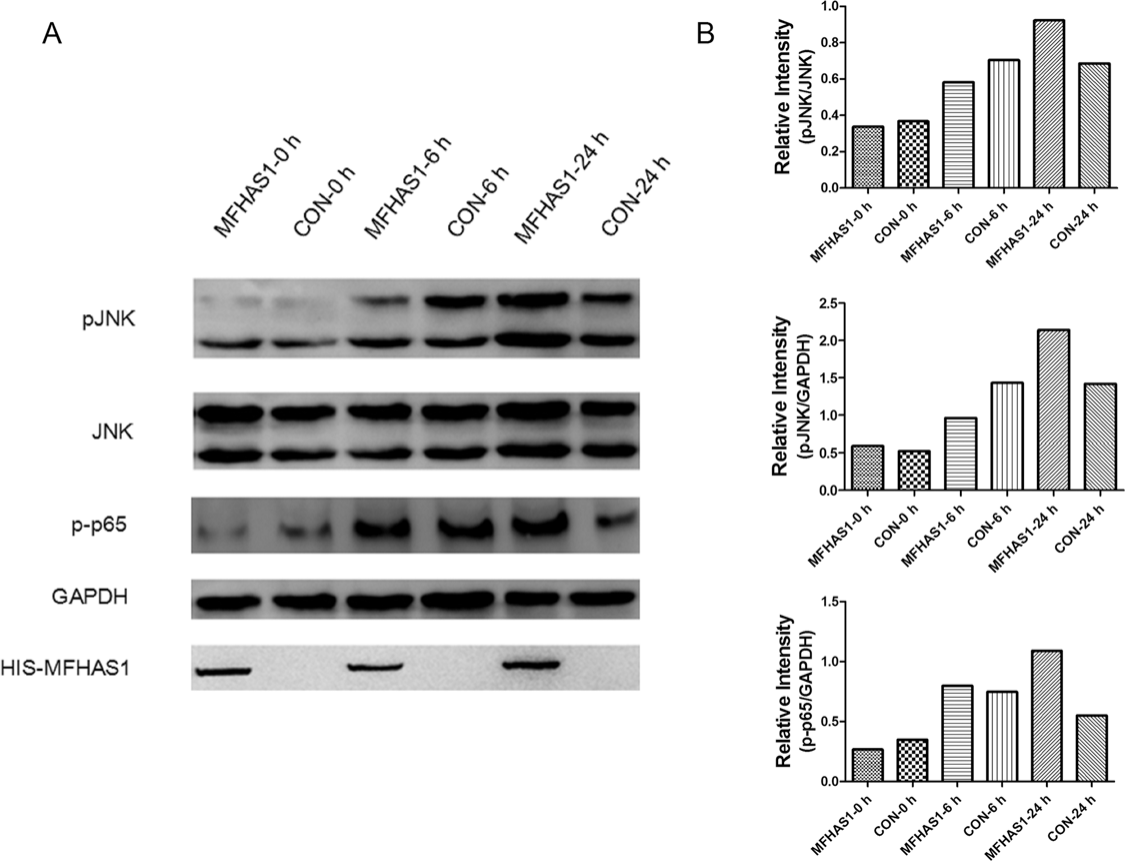
MFHAS1 activates pJNK in a time-dependent manner. (A) RAW 264.7 macrophages were transfected with HIS-MFHAS1 or pCDH empty vector plasmid, and 24 h post-transfected cells were untreated or treated with 10 ng/mL Pam3CSK4 for 6 h or 24 h. Cells were collected and pJNK, JNK, p-p65, GAPDH and HIS-MFHAS1 protein levels were determined by western blotting. (B) Quantified data of the pJNK and p-p65 levels. Levels of pJNK normalized to JNK levels, and pJNK/p-p65 normalized to GAPDH were shown.

However, following 6 h stimulation through TLR2 by Pam3CSK4, the levels of pJNK expression in 264.7-MFHAS1 cells were lower than that in the control. MFHAS1 inhibits the phosphorylation of JNK after 6 h TLR2 stimulation. After 24 h TLR2 stimulation, pJNK expression levels were found to be higher in 264.7-MFHAS1 cells than that in the control once again. MFHAS1 activates the phosphorylation of JNK after 24 h TLR2 stimulation. The phosphorylation of NF-κB p65 was also increased in 264.7-MFHAS1 cells than that in the control following 24 h TLR2 stimulation through Pam3CSK4 (Fig 7).

## Discussion

Sepsis, which is a huge and expensive medical problem worldwide, can lead to septic shock, multiple organ dysfunction syndrome and death. Physicians and researchers are actively engaged in finding biomarkers or biomodulators in sepsis. Our study shows that MFHAS1’s activity is associated with sepsis in humans and MFHAS1 levels increase during immune response. MFHAS1 is an intracellular protein. Since the expression of *MFHAS1* was increased in PBMC from patients with sepsis in this article, the elevation of blood MFHAS1 may because it gets released during cell necrosis in sepsis. Biomarkers such as IL-6 and CRP etc. are widely studied in the diagnosis and treatment of sepsis. Our results suggest that the role of MFHAS1 in sepsis and its potential utility in the management of this condition must be investigated further.

The essence of sepsis is inflammation. TLR signaling acts synergistically in the initiation of the innate immune response to bacterial infection during sepsis. Our study investigated the effect of MFHAS1 on the TLR2 signaling pathway, and its difference from TLR4. We used Pam3CSK4 to stimulate the TLR2 signaling pathway in HEK 293 cells and RAW 264.7 cells, as Pam3CSK4 is a specific ligand for TLR2. Interestingly, we find that MFHAS1 exerts dual effects on the TLR2 signaling pathway in both HEK 293 cells and RAW 264.7 cells. HEK 293 cells lack TLRs. When we transfected HEK 293 cells with TLR2 expression plasmid, the reaction of downstream signaling was due to the activation of TLR2. Therefore, we can confirm that TLR2 is responsible for the dual effects of MFHAS1. We also used shRNA to verify MFHAS1’s effect on TLR2. Since our aim was to study the human *MFHAS1* gene, we used human shRNA to knockdown *Mfhas1* in RAW 264.7 cells. The RAW 264.7 cell, which is of murine origin, is an adherent cell that can be transfected more easily, unlike the human macrophage U937 and THP-1 suspended cells. We tested the efficiency of shMFHAS1 in this study by RT-PCR and western blotting, and results obtained were found to support that shMFHAS1 was successful in the RAW 264.7 cell. Using this shMFHAS1 technique in the RAW 264.7 macrophage, we find that MFHAS1 has an inhibitory effect on TLR2 signaling pathway and inflammation at the first 6 hours, and a stimulatory effect after 24 hours. This conforms to the results obtained from HEK 293 cells. However, we have not got the same effect of MFHAS1 on TLR4 using the same shMFHAS1 technique. Bone [37] has introduced the term “compensatory anti-inflammatory response syndrome” (CARS), reflecting immunoparalysis in septic patients in contrast to the systemic inflammatory response syndrome (SIRS). SIRS was thought to be followed by CARS. However, more and more studies suggest that SIRS and CARS appear alternately, that is immunological variables behave in a mixed and time-dependent manner [38–41]. MFHAS1 may represent one of the factors that changes in the different stages of sepsis through TLR2, although the time span of the inhibitory effect of MFHAS1 is short. Twenty-four hours after activation of TLR2, MFHAS1 stimulates the production of inflammatory cytokines, resulting in pro-inflammatory effects.

Ng et al. showed that the siRNA knockdown of *Mfhas1* in RAW 264.7 macrophages strongly enhanced IL-6 production following LPS and polyI:C stimulation. Our previous studies (unpublished) of the TLR4 signaling pathway indicated similar results, suggesting that MFHAS1 might play a negative regulatory role in the TLR4 signaling pathway. These results differ slightly from this study. Although TLR2 and TLR4 share similar downstream pathways, MFHAS1 affects TLR2 signaling pathway in a different way from TLR4, according to this study. Moreover, Ng et al’s study did not measure the NF-κB, AP-1 luciferase activity, or IL-6, TNF-α production at 24 h following TLR activation. They only measured 6 h data. This may explain the different results obtained. The difference in ligands used in this study, compared to the other studies referred to, may contribute another reason.

**Fig 8:**
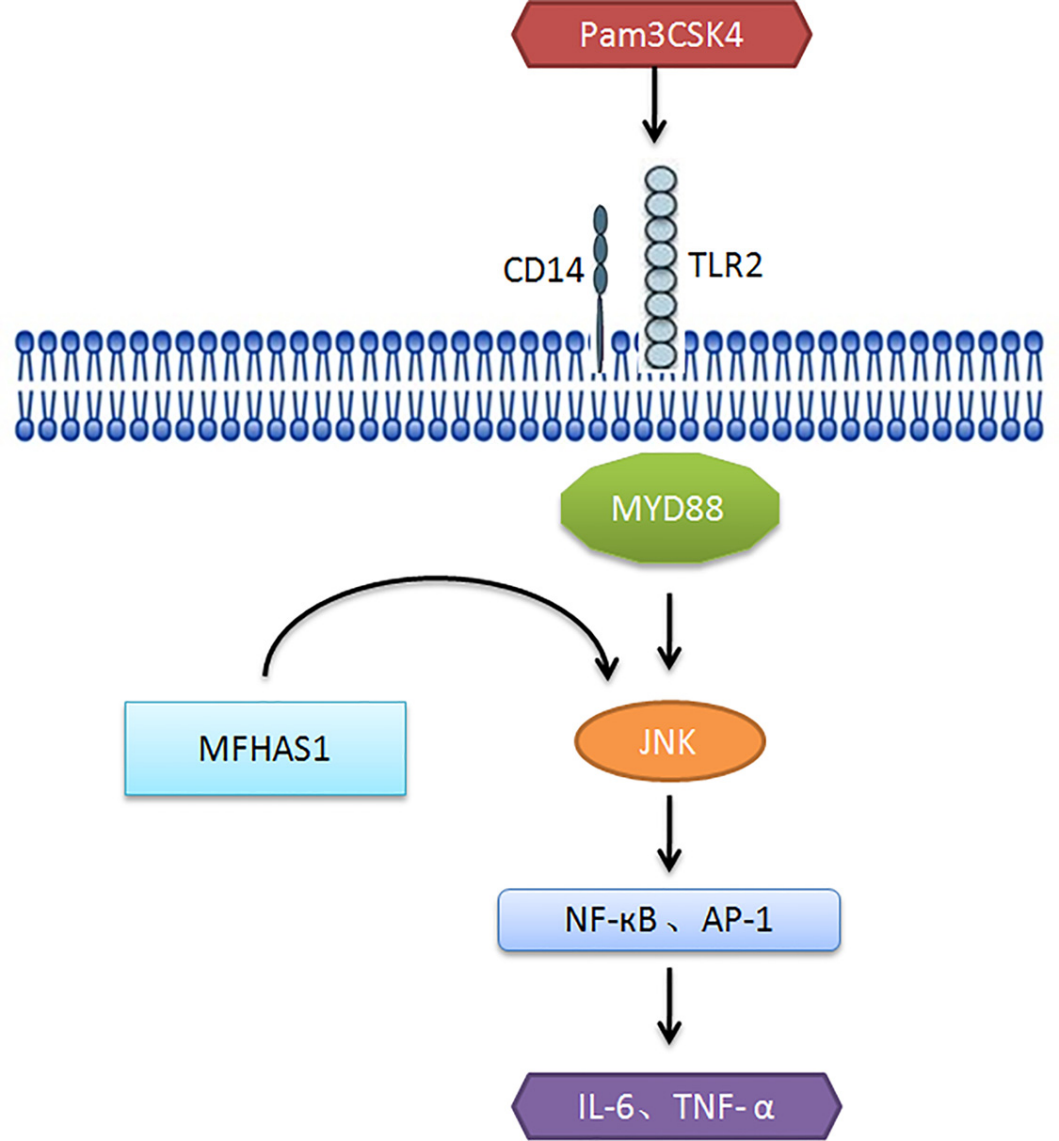
Model of the effect of MFHAS1 on TLR2 signaling pathway in response to TLR2 stimulation for 6–24 h.

In order to further investigate the TLR2 signaling pathway and the dual effect of MFHAS1, we focused on the downstream signaling pathway of TLR2 affected by MFHAS1. A few signaling cascades may be triggered when TLR2 is stimulated. In this study, MFHAS1 was observed to stimulate the phosphorylation of JNK and p38 immediately after TLR2 activation. However, while TLR2 was stimulated for 6 h, the effect of MFHAS1 on TLR2 was inhibitory, compared to the control. MFHAS1 was found to inhibit the phosphorylation of JNK, and the production of NF-κB and IL-6 at 6 h TLR2activation. The inhibitory effect of MFHAS1 on TLR2 does not continue. After 24 h TLR2 activation, MFHAS1 was found to stimulate the phosphorylation of JNK, NF-κB p65 and the production of NF-κB and IL-6. Therefore, we propose a pathway through which MFHAS1 affects TLR2 signaling cascade after 6–24 h TLR2 stimulation: TLR2 ligands (Pam3CSK4 in this article) activate TLR2, and then MFHAS1 activates the JNK signaling pathway, which induces the transcription factors NF-κB and AP-1, resulting in the elevation of inflammatory cytokines IL-6 and TNF-α (Fig 8).As to the pathway affected by MFHAS1 within 6 h TLR2 stimulation, and the reason why the stimulatory effect of MFHAS1 does not sustain in the first 6 h, it needs further study. Other pathways may involve in the effect of MFHAS1 on TLR2 signaling pathway.

IFN-β mRNA induction, stimulated by TLR2, is regulated by IRF7 and NF-κB. TLR2-mediated IRF7 activation requires endocytosis. We found that MFHAS1 did not affect transcription factor IRF-7 and IFN-β production in this study, suggesting that MFHAS1 does not have any effect on the endocytosis of TLR2 during inflammation. In this study, we find that CD14 can assist TLR2 to further enhance the activation of NF-κB, AP-1, and IRF7.These results conform to the effect of CD14, which acts as a TLR-associated molecule.

In accordance with the effect of MFHAS1 on TLR2 signaling pathway, directly targeting MFHAS1 may affect the inflammatory reaction. MFHAS1 has dual effects on TLR2 mediated inflammation, which makes it a target of interest in the design of new therapeutics to restore immune balance in infectious and inflammatory diseases. Further research should be focused on whether MFHAS1 has the same effect on TLR2, using other ligands like bacterial lipopeptides, and animal experiments should be performed to further verify the effect of MFHAS1 on the TLR2 signaling pathway, and its effect on inflammation and sepsis.

In conclusion, through TLR2, MFHAS1 has inhibitory effect on the TLR2 signaling pathway and inflammation at the first 6 hours, and then has stimulatory effects after 24 hours through JNK/NF-κB/AP-1 signaling pathway. In the context of sepsis, research efforts have been directed toward molecules with the potential to affect inflammatory cytokines such as IL-6 and TNF-α, in order to alleviate SIRS during sepsis. Given its dual effects on TLR2 signaling pathway and inflammation, MFHAS1 might be a suitable novel therapeutic tool to achieve immune balance in sepsis.

## Supporting Information

S1 Fig The transfection efficiency does not differ significantly between groups. HEK 293 cells and 293-MFHAS1 cells were transiently transfected with TLR2 or TLR2/CD14 expression plasmids for luciferase activity test. Cells were collected and lysed with cell lysis buffer added with PMSF. The expression level of TLR2 was evaluated by western blotting. A. In the NF-κB-dependent luciferase activity test, cells were treated with Pam3CSK4 for 6 h. B. In the NF-κB-dependent luciferase activity test, cells were treated with Pam3CSK4 for 24 h. C. In the IRF-7 luciferase activity test, cells were treated with Pam3CSK4 for 24 h.
(PDF)

S1 Table Primers used in this study. (PDF)
