# Supplementary material for: MFHAS1 Is Associated with Sepsis and Stimulates TLR2/NF-κB Signaling Pathway Following Negative Regulation
Source: PLoS One. 2015 Nov 24;10(11):e0143662. doi: 10.1371/journal.pone.0143662 (PMC4658032; doi:10.1371/journal.pone.0143662)
Supplement: S1 Fig — HEK 293 cells and 293-MFHAS1 cells were transiently transfected with TLR2 or TLR2/CD14 expression plasmids for luciferase activity test. Cells were collected and lysed with cell lysis buffer added with PMSF. The expression level of TLR2 was evaluated by western blotting. A. In the NF-κB-dependent luciferase activity test, cells were treated with Pam3CSK4 for 6 h. B. In the NF-κB-dependent luciferase activity test, cells were treated with Pam3CSK4 for 24 h. C. In the IRF-7 luciferase activity test, cells were treated with Pam3CSK4 for 24 h. (PDF) [file pone.0143662.s001.pdf]

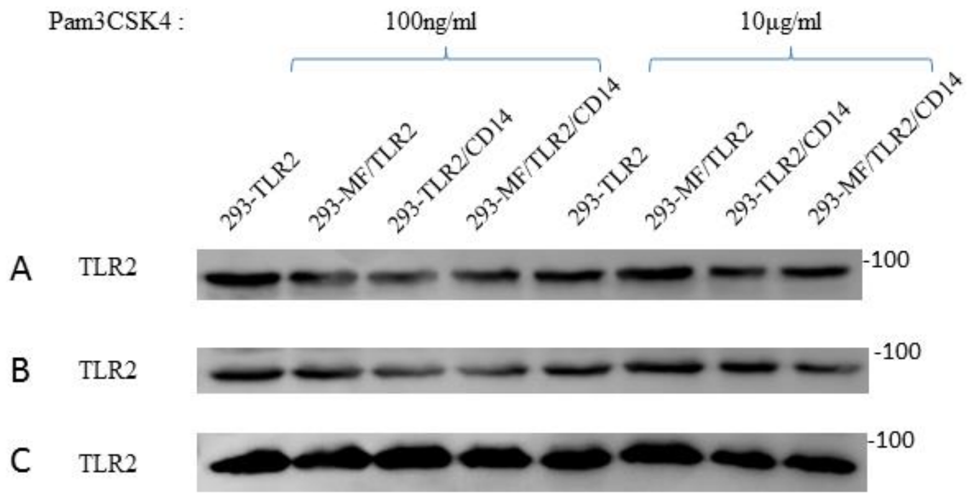

S1 Fig. The transfection efficiency does not differ significantly between groups.

HEK 293 cells and 293-MFHAS1 cells were transiently transfected with TLR2 or TLR2/CD14 expression plasmids for luciferase activity test. Cells were collected and lysed with cell lysis buffer added with PMSF. The expression level of TLR2 was evaluated by western blotting. A. In the NF-κB-dependent luciferase activity test, cells were treated with Pam3CSK4 for 6 h. B. In the NF-κB-dependent luciferase activity test, cells were treated with Pam3CSK4 for 24 h. C. In the IRF-7 luciferase activity test, cells were treated with Pam3CSK4 for 24 h.
