# Supplementary material for: MFHAS1 Is Associated with Sepsis and Stimulates TLR2/NF-κB Signaling Pathway Following Negative Regulation
Source: PLoS One. 2015 Nov 24;10(11):e0143662. doi: 10.1371/journal.pone.0143662 (PMC4658032; doi:10.1371/journal.pone.0143662)
Supplement: S1 Table — (PDF) [file pone.0143662.s002.pdf]

S1 Table. Primers used in this study.

Genes	Sequence (5'–3')
hIL6-F	agtcctgatccagttcctgc
hIL6-R	aagctgcgcagaatgagatg
hGAPDH-F	acaactttggtatcgtggaagg
hGAPDH-R	gccatcacgccacagtttc
hIFN-β-F	atgaccaacaagtgtctcctcc
hIFN-β-R	ggaatccaagcaagttgtagctc
mIL6-F	ccggagaggagacttcacag
mIL6-R	ttctgcaagtgcatcatcgt
mMfhas1-F	gagatcatctgccccaagaa
mMfhas1-R	ttctgtgcttctcaccaacg
hMFHAS1-F	gcctcatgctagacaacaacg
hMFHAS1-R	gctggttgcgactaaggtagag
mβ-actin-F	tccctggagaagagctacga
mβ-actin-R	agcactgtgttggcgtacag
